# Transcriptomic and metabolomic joint analysis reveals distinct flavonoid biosynthesis regulation for variegated testa color development in peanut (*Arachis hypogaea* L.)

**DOI:** 10.1038/s41598-021-90141-6

**Published:** 2021-05-21

**Authors:** Mengdie Hu, Jiawei Li, Mingyu Hou, Xiaoqing Liu, Shunli Cui, Xinlei Yang, Lifeng Liu, Xiaoxia Jiang, Guojun Mu

**Affiliations:** 1 North China Key Laboratory for Crop Germplasm Resources of Education Ministry, College of Agronomy, Hebei Agricultural University, Baoding, 071001 China; 2 Hebei Yiyuan Ecological Agriculture Technology Co, Ltd., Baoding, 074200 China

**Keywords:** Molecular biology, Transcriptomics, Plant sciences, Plant molecular biology, Secondary metabolism

## Abstract

Peanut is one of the important oil and economic crops, among which the variegated testa peanut is a unique member. The molecular mechanisms underlying the pigment synthesis in variegated testa are still unclear. Differentially expressed genes (DEGs) in the flavonoid metabolism pathway in pigmented areas indicated that there were 27 DEGs highly related to the synthesis of variegated testa color among 1,050 DEGs. Of these 27, 13 were up-regulated and 14 were down-regulated, including 3 *PALs*, 1 *C4H*, 2 *CHSs,* 1 *F3H,* 1 *F3'H,* 2 *DFRs,* 2 *LARs,* 2 *IAAs,* 4 *bHLHs,* and 9 *MYBs*. GO (Gene Ontology) analysis indicated that DEGs were similarly enriched in three branches. KEGG (Kyoto Encyclopedia of Genes and Genomes) analysis suggested flavonoid biosynthesis is the most direct metabolic pathway for the synthesis of testa variegation. The liquid chromatography–tandem mass spectrometry (LC–MS/MS) results showed that cyanidin and delphinidin were the primary metabolites that caused the color differences between the pigmented and the non-pigmented areas. Through the verification of 20 DEGs via qPCR, the results were consistent with transcriptome sequencing in four comparison groups. The results in this study lay the foundation for revealing the molecular regulation mechanisms of flavonoid synthesis in variegated testa peanut.

## Introduction

Peanut (*Arachis hypogaea* L.) is the primary oil and economic crop that occupies an important position in the world for grain, oil, and food. It is widely planted in developing countries in Asia, Africa, and South America, as well as some developed countries including the United States and Australia^1^. Peanut testa contains a variety of biologically active substances, which are important raw materials for cosmetics and medicinal health products^2^. The variegated testa peanut is a unique member of the peanut family. The variegated testa is composed of pigmented and non-pigmented areas, with the pigmented areas including purple, red, pink, and other colors. The variegated testa is a unique agronomic trait, which is combined with other traits such as high sugar and high oleic acid content in breeding for peanut quality. The distinctive phenotype of the trait facilitates planting and breeding. In addition, variegated testa peanuts enrich the peanut germplasm resources in China. Different types and content of anthocyanins will cause peanut testa to appear different colors. Anthocyanins also have many pharmacological functions in anti-oxidation, anti-inflammatory, eyesight protection, antibacterial, and other aspects^3^.

The development of different colors on the same variegated testa is an interesting natural phenomenon within the same genetic background. However, the molecular mechanism of pigment synthesis in the testa is still unclear. Plant anthocyanin metabolism is a complex process that included chalcone synthase (CHS), chalcone isomerase (CHI), dihydroflavonol-3-hydrogenase (F3H), dihydroflavonol-3′-hydrogenase (F3′H), dihydroflavonol-3′-5′hydrogena, dioxanonol-4-reductase (DFR), anthocyanidins synthase (ANS), and many other key enzymes^4^. Meanwhile, basic helix-loop-helix (bHLH), v-myb avian myeloblastosis viral oncogene homolog (MYB), WD40 protein, and other transcription factors can also regulate the expression of structural genes in the plant anthocyanin metabolic pathway^5^. The first enzyme (CHS) in anthocyanin synthesis can affect the shade of plant color^6^ and the flower color can change from purple to pink or even white if the *CHS* antisense gene is transformed in *Petunia hybrida*^7^. The color differentiation of tea tree (*Camellia sinensis*) buds and leaves are catalyzed by *DFR* and *ANS* from three main anthocyanidins^8–11^. MYB and bHLH are important regulatory genes in the anthocyanin synthesis pathway of *Petunia hybrida*^12^. Overexpression of *Rs-MYB1* in petunia activates the expression of *ANS* and *DFR* in the anthocyanin synthesis process and increases the anthocyanin content^13^. The regulation of anthocyanin synthesis by bHLH transcription factors has been widely investigated in *Arabidopsis*, snapdragon, chrysanthemum, begonia, and other plants^14–16^. The degree of coloration often only requires the difference in the expression of one or several key anthocyanin genes between the pigmented and the non-spotted areas and does not require all of the anthocyanin genes to have an obvious difference in expression^17^. For example, the formation of white star-shaped spots in *P. hybrida* is caused by the inhibition of *CHS-A* expression^18^. The large purple spots in *Phalaenopsis stuartiana* are primarily due to the specific expression of *DFR* in the spot areas^19^, while other spots are caused by the expression difference of two or more anthocyanin genes. For example, the color develops if the expression of *OgCHI* and *OgDFR* is suppressed in *Oncidium hybridum*^20^. Xia et al*.* found that the up-regulation of *DFR* in Chinese peanut variety ZH9 is beneficial to the promotion of dihydroflavonol synthesis in the anthocyanin and flavonol synthesis pathways^16^. Li et al*.* studied four types of peanut testa with different colors 40 and 50 days after anthesis and found that *CHS* was expressed in large quantities, promoting anthocyanin synthesis. In addition, the expression of *DFR* was significantly related to the testa color^21^. Wan et al*.* adopted transcriptome sequencing to analyze the color accumulation of the testa of the pink peanut variety ZH16, showing that the expression of PAL, 4CL, CHS, CHI, and other enzymes increased, while the expression of ANR and LAR decreased^22^. Li et al*.* found that the relative expression level of *F3H* in purple peanut variety FH01 is positively correlated with anthocyanin content^23^. One reason for the lack of research on the pigment synthesis and molecular mechanism of the variegated peanut testa is related to the scarcity of the research materials.

Transcriptome sequencing is a powerful technique that can identify almost all transcripts and gene sequences of a specific cell or tissue of a certain species in a certain state and can be used to study gene expression, gene function, structure, alternative splicing, and forecasting new transcripts. Ye et al*.* adopted this technology to screen out 15 key structural genes involved in the metabolism of peach skin anthocyanins, including the upstream phenyl propionic acid metabolism pathway genes, such as *PAL*, *C4H,* and *4CL*, and the late anthocyanin metabolism pathway genes, such as *CHS*, *CHI*, *F3H*, *F3'H*, *DFR*, *LAR*, and *ANR*^24^. Liu et al*.* found that the expression levels of anthocyanin biosynthesis genes *PAL* and *C4H* and transcription factor RsMYB1 and RsTT8 were significantly down-regulated during the development in a radish variety “Xin Li Mei”^25^. Jia et al*.* concluded that ANS is the key gene determining kiwifruit anthocyanin synthesis via transcriptome sequencing^26^. Jin et al*.* analyzed transcriptomes of differently pigmented *Cineraria* flowers (white and yellow), which were not able to accumulate anthocyanins due to mutations in the coding regions of specific genes that cause variations in color development^27^. Transcriptome sequencing has been widely used to study various species including apple^28^, grape^29^, kiwifruit^30^, and soybean^31^.

In this study, we used transcriptomics to quantitatively analyze the differential genes related to the flavonoid synthesis pathway. Different parts of the same peanut testa tissue were used as experimental materials. The differences in metabolites were compared between the pigmented and non-pigmented areas at the same developmental stage, and the same testa areas (pigmented and non-pigmented) at different developmental stages were also compared. The results show different relative contents of metabolites, and also demonstrate the changes in metabolites between the pigmented and non-pigmented areas. The relative content of flavonoids in different parts of the variegated testa at different developmental stages was determined with metabonomics by screening out key genes involved in flavonoid synthesis in peanut. This paper initially revealed the molecular regulation mechanism of flavonoid synthesis in the variegated testa of peanut and facilitate further in-depth studies in peanut functional genomics.

## Results

### Characterization of variegated testa by stereomicroscope

**Figure 1:**
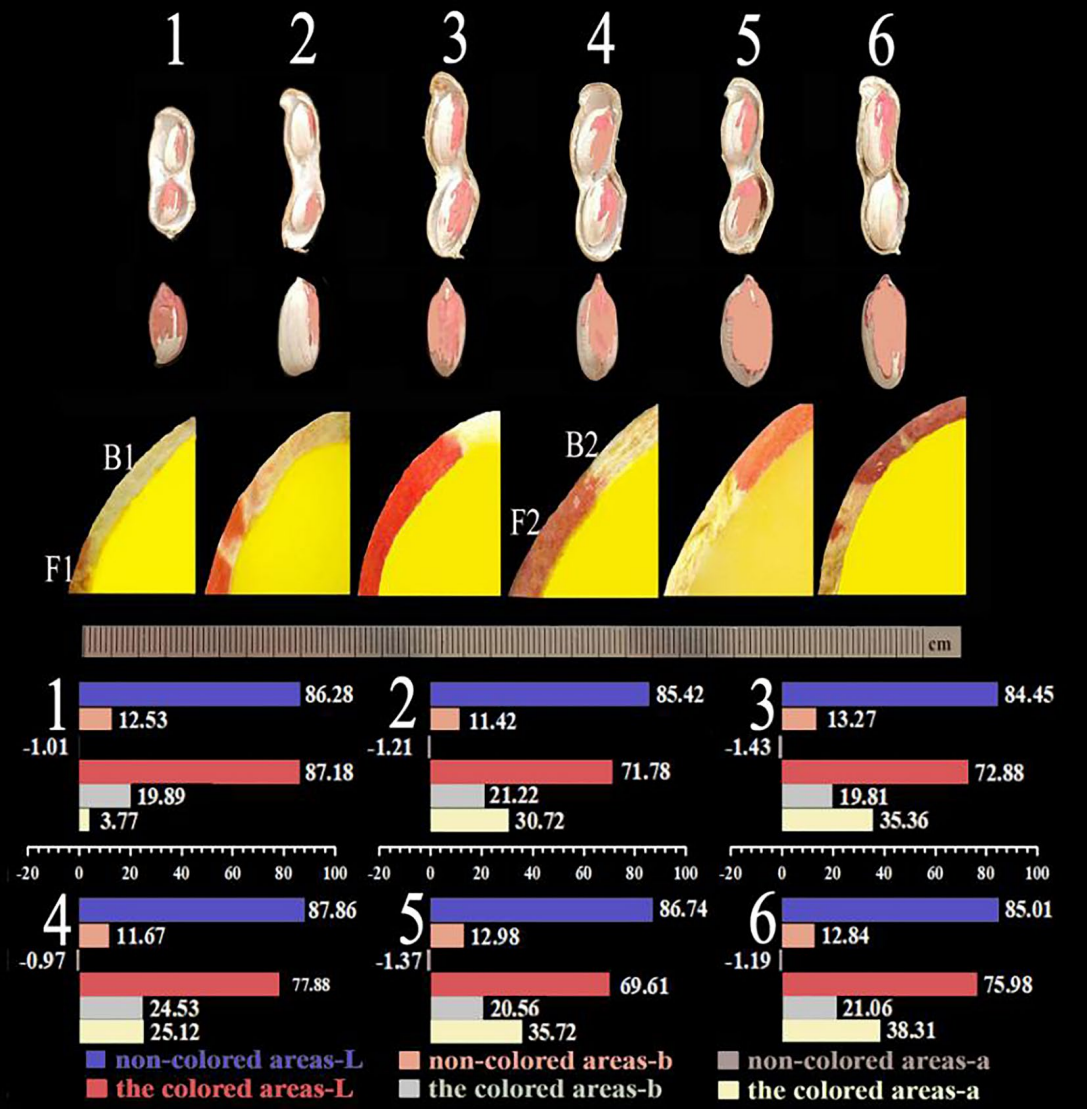
Phenotype and chromatic changes in the synthesis of color in the variegated peanut testa: Numbers 1–6: day at flowering and needling (DAF) 30, 35, 40, 45, 50, and 55 days. F1 and F2 represent the sample names of the pigmented areas at DAF30 and DAF45, respectively. B1 and B2 represent the sample names of the non-pigmented areas at DAF30 and DAF45, respectively. Bar colors represent different values of “a”, “b”, and “L”. The absorbance value “a” (“a” value) represents red and green; the absorbance value “b” (“b” value) represents yellow and blue; and the “L” value represents lightness.

Pigmentation in the pigmented areas (F) of the variegated peanut testa was 30 days after flowering and needling (DAF30), which was the active period of related gene expression. The “red, green” (a) and “yellow, blue” (b) values of the non-pigmented areas (B) were lower than in the pigmented areas during testa development. The “lightness” value (L) in the non-pigmented areas was higher than in the pigmented areas, which was possibly due to the white color of the testa in non-pigmented areas. As the pod matured 45 days after flowering and needling, the color of the variegated testa stabilized. The values of “a” and “b” in the pigmented areas changed slightly and “L” increased to 12.85% (Fig. 1, Table S1). The pigmented and non-pigmented areas sampled were located on the same testa with identical genetic backgrounds, which is ideal for the study of DEGs in variegated testa pigmentation.

### Measurement of flavonoids

**Figure 2:**
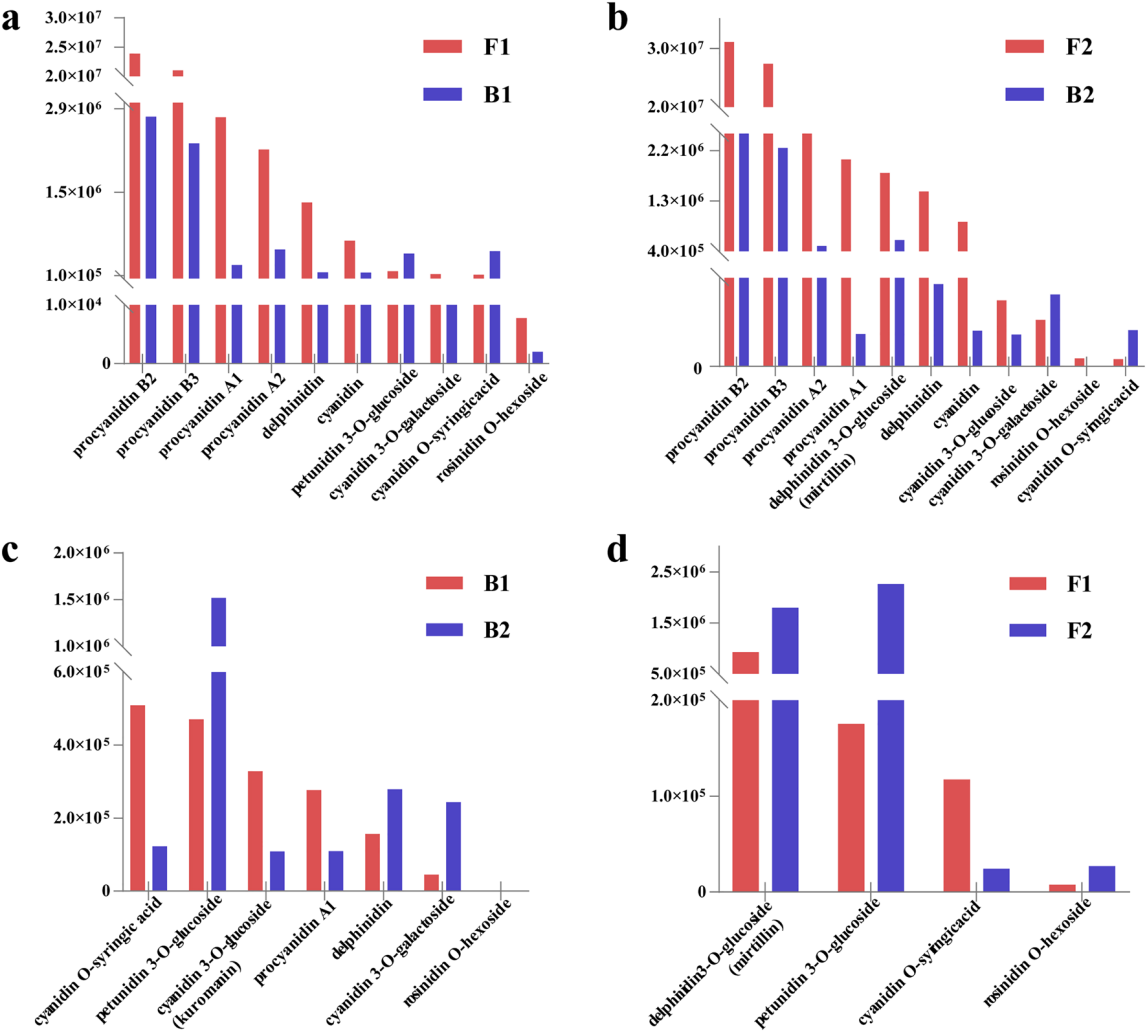
Types and relative contents of flavonoids in different areas of peanut testa at different developmental stages. (**a**, **b**) represent the type and content differences of metabolized flavonoids between the pigmented and non-pigmented areas after DAF30 and DAF45, respectively. (**c**) represents the different metabolized flavonoid types and content in the non-pigmented areas after DAF30 and DAF45. (**d**) represents the different metabolized flavonoid types and content in the pigmented areas at DAF30 and DAF45. The horizontal axis represents the types of flavonoids in the variegated testa and the vertical axis represents the peak areas value determined by the LC–MS/MS detection platform.

The pigmented (F1 and F2) and non-pigmented (B1 and B2) areas were compared at DAF30 and DAF45. In the comparison of F1–B1, the total content of the ten different metabolites measured in the pigmented areas was 7.24 times higher than that in the non-pigmented areas. The content of procyanidin A1, A2, B2, B3, delphinidin, cyanidin, cyanidin 3-O-galactoside, and rosinidin O-hexoside in the pigmented areas increased by 185.40–895.58%, while the relative content of petunidin 3-O-glucoside and cyanidin O-syringic acid in the pigmented areas were lower than in the non-pigmented areas, by 62.70–76.92% (Fig. 2a). In the comparison of F2–B2, the total content of the 11 different metabolites measured in the pigmented areas was 9.95 times higher than that in the non-pigmented areas. The relative content of procyanidin A1, A2, B2, B3, delphinidin 3-O glucoside, delphinidin, cyanidin, cyanidin 3-O-glueoside, rosinidin O-hexoside in the pigmented areas increased by 106.54–1759.77%. However, compared with the pigmented areas, the relative content of cyanidin 3-O-galactoside and cyanidin O-syringic acid in the non-pigmented areas was increased by 35.25–80.35% (Fig. 2b). The relative content of cyanidin and delphinidin are highest in colored metabolites in the comparison F1–B1, which is same as F2–B2. In the comparison of B1–B2, at DAF45, the total content of the seven different metabolites measured was 1.33 times higher than that at DAF30 in the non-pigmented areas. The relative content of petunidin 3-O-glucoside, delphinidin, cyanidin 3-O-galactoside at DAF45 increased by 43.73–81.62%, while the relative content of cyanidin O-syringic acid, cyanidin 3-O-glucoside, procyanidin A1, rosinidin O-hexoside at DAF45 decreased by 152.39–313.82% compared with DAF30 (Fig. 2c). The metabolites between F1 and F2 were almost identical. At DAF45, the total content of the four different metabolites measured was 3.35 times higher than that at DAF30 in the pigmented areas. The relative content of delphinidin 3-O-glucoside, petunidin 3-O-glucoside, and rosinidin O-hexoside at DAF45 was 48.44–92.25% higher than at DAF30, while the relative content of cyanidin O-syringic acid at DAF45 was lower by 386.24% compared to DAF30 (Fig. 2d, Table S2).

### RNA sequencing

Transcriptome sequencing was performed on F1, B1, F2, and B2 of the variegated peanut (accession VG-01). The adapter sequences and low-quality read sequences were all removed from the sequencing data. A total of 64.33 Gb clean data were obtained, and the clean data of each sample reached 6.53 Gb. The base recognition error rate was about 0.02%, the Q20 value of the obtained sequence was above 97.45%, the Q30 base percentage was above 93.23%, and the GC content was above 43.90% (Table S3).

### Detection of DEGs

**Figure 3:**
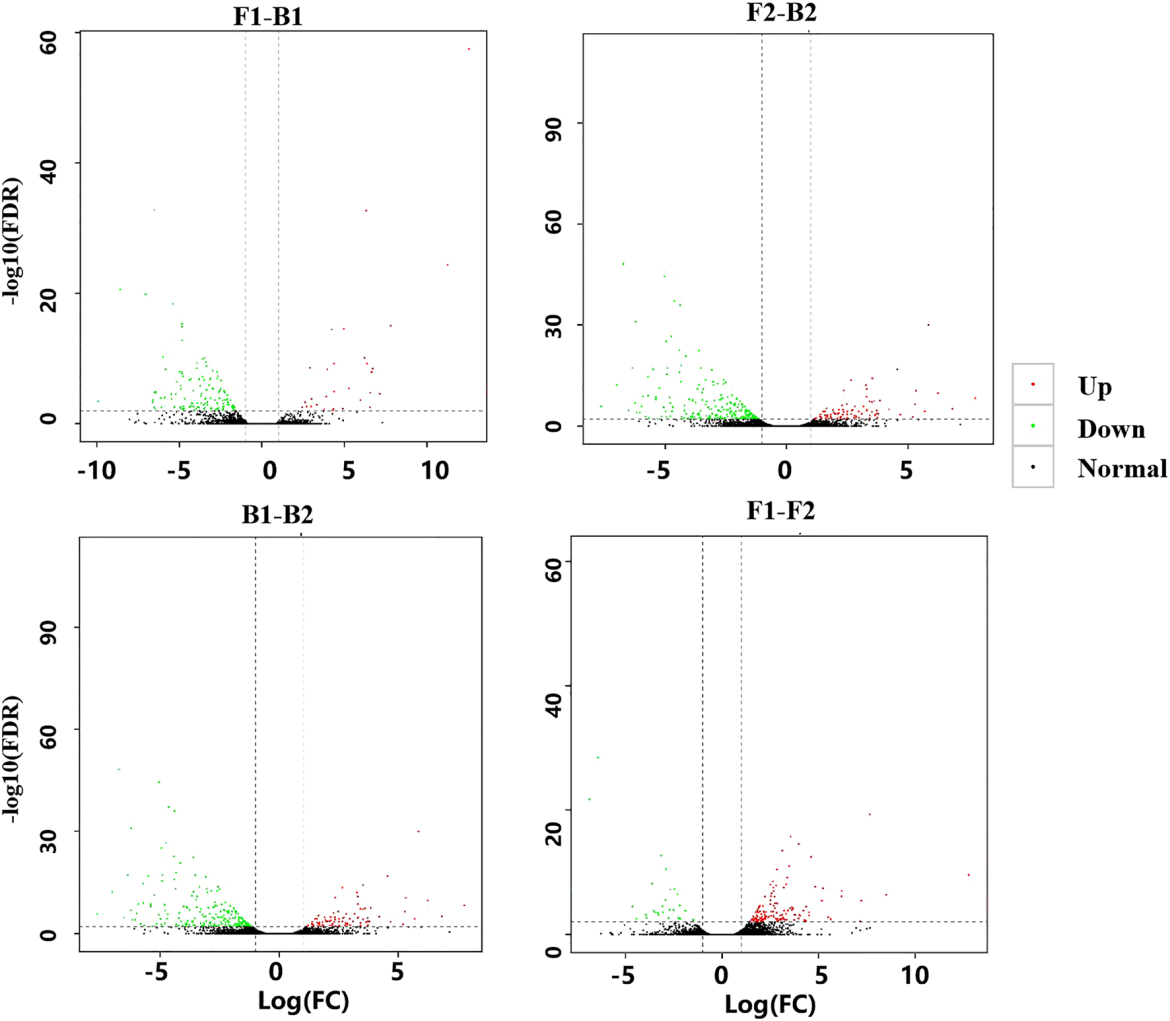
Analysis of the volcano map of different genes in various parts of the peanut testa at different developmental stages. The abscissa indicates the multiple of difference, and the ordinate indicates − log10 (q-value). Red dots indicate up-regulation of gene expression, green dots indicate down-regulation of gene expression, and black dots indicate that there is no significant difference in gene expression.

With reference to the tetraploid reference genome of peanut and hierarchical clustering analysis, a total of 214 differential genes in F1–B1 (F1 is the control group and B1 is the comparison group) were identified, of which 53 were up-regulated and 161 were down-regulated. There were 348 differential genes identified in F2–B2 (F2 is the control group and B2 is the comparison group), including 97 up-regulated and 251 down-regulated. There was a total of 152 differential genes in B1–B2 (B1 is the control group and B2 is the comparison group), including 82 up-regulated and 70 down-regulated genes, and 213 differential genes in F1–F2 (F1 is the control group and F2 is the comparison group), with 169 up-regulated and 44 down-regulated (Fig. 3, Table S4).

### GO enrichment analysis of DEGs

**Figure 4:**
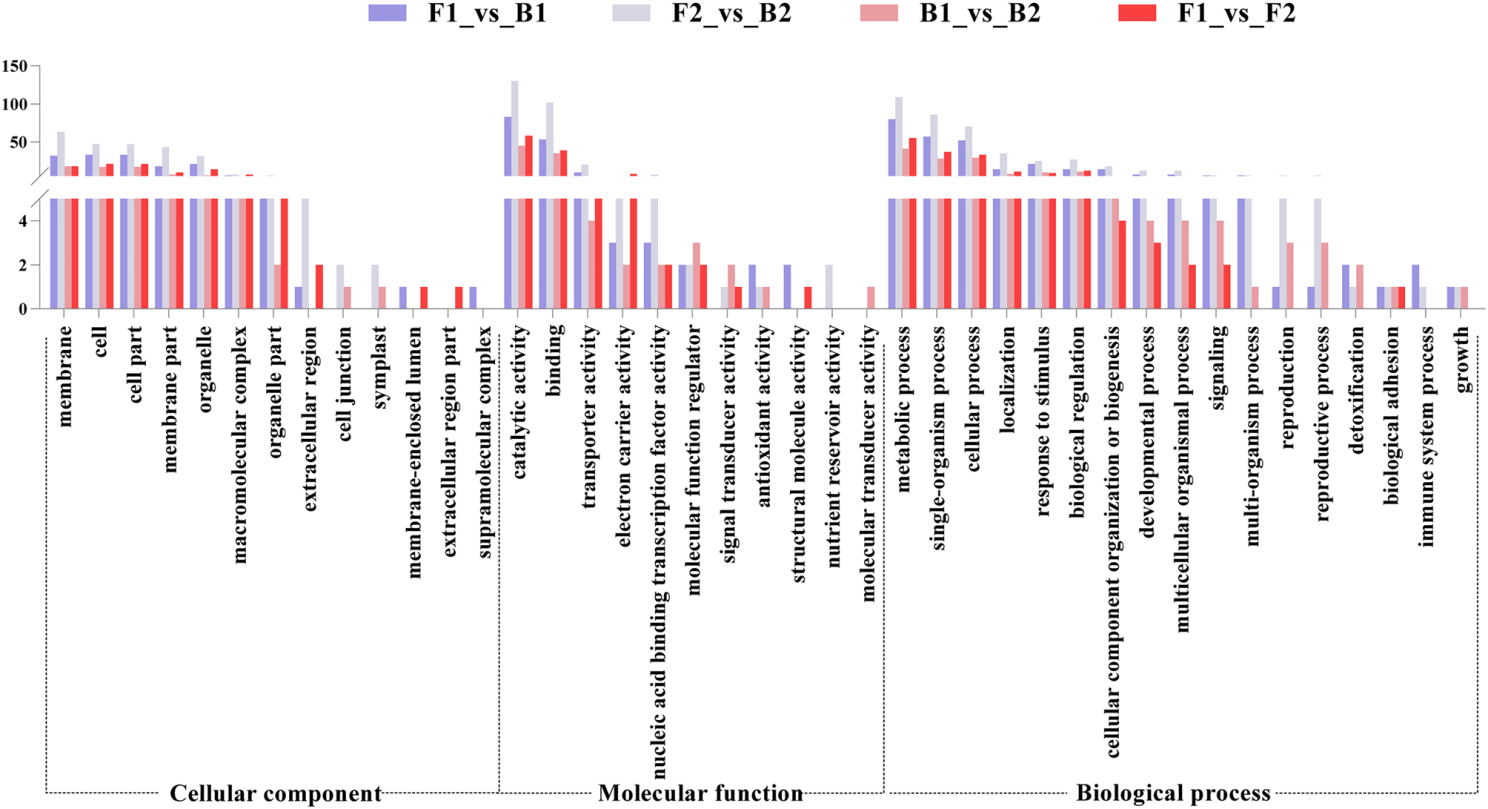
GO functional enrichment classification of variegated peanut testa transcriptome. Differently colore bars represent four comparison groups. The y-axis represents the number of genes mapped to the indicated GO term and the x-axis represents each GO term. GO terms with an adjusted p-value of < 0.05 were considered significantly enriched.

1050 DEGs (accounting for 82.48% of all differential genes) were compared with GO public databases of tetraploid cultivated peanut and diploid wild peanut, respectively. The comparison results of the tetraploid reference genome showed that F1–B1, F2–B2, F1–F2, and B1–B2 comparison groups were all enriched in 51 GO entries and the diploid genome comparison showed that four comparison groups were all enriched in 41 GO entries (Table S5). Following comparative analysis, the enrichment items in three branches of four comparison groups were horizontally similar. Analysis of the tetraploid cultivar transcriptome data revealed that the enriched items in the cellular components category were membrane, and membrane part, the molecular function were enriched in catalytic activity, binding, and transporter activity. In addition, biological processes showed that DEGs enriched in metabolic, single-organism, and cellular processes (Fig. 4).

### COG enrichment analysis of DEGs

**Figure 5:**
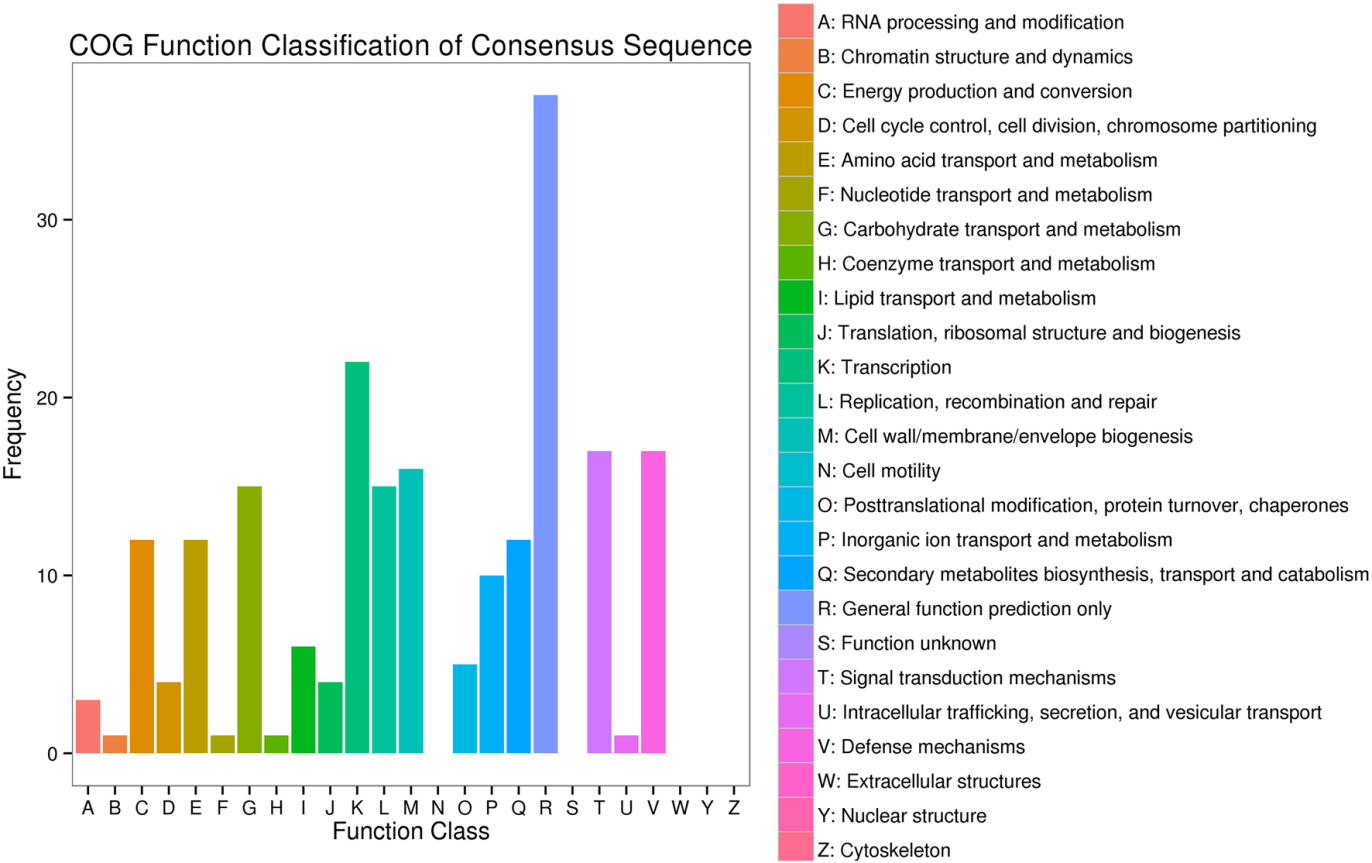
COG functional classification compared with the tetraploid peanut reference genome. A: RNA processing and modification; B: Chromatin structure and dynamics; C: Energy production and conversion; D: Cell cycle control, cell division, chromosome partitioning: E: Amino acid transport and metabolism; F: Nucleotide transport and metabolism; G: Carbohydrate transport and metabolism; H: Coenzyme transport and metabolism; I; Lipid transport and metabolism; J: Translation, ribosomal structure and biogenesis; K: Transcription; L: Replication, recombination and repair: M: Cell wall/membrane/envelope biogenesis; N: Cell motility; O: Posttranslational modification protein turnover, chaperones; P: Inorganic ions transport and metabolism; Q: Secondary metabolites biosynthesis, transport and catabolism; R: General functional prediction only; S: Function unknown; T: Signal transduction mechanism; U: Intracellular trafficking, secretion, and vesicular transport; V: Defense mechanisms; W: Extracellular structures; Y: nuclear structure; Z: Cytoskeleton.

The annotated genes were compared with the COG database and it was found that F1–B1 contained the largest DEGs proportion (R-General functional prediction only), accounting for 12.61%, followed by Transcription, accounting for 6.54%. Amino acid transport and metabolism accounted for 5.61%. Energy production and conversion, and Defense mechanisms accounted for 4.67%. F2–B2 had the largest DEGs proportion (R-General functional prediction only), accounting for 10.63%, followed by Transcription, accounting for 6.32%. Amino acid transport and metabolism accounted for 5.61%. Energy production and conversion, and Defense mechanisms accounted for 4.89%. DEGs in B1–B2 contained the largest DEGs proportion (R-General functional prediction only), accounting for 5.92%, followed by Transcription, accounting for 4.61%. The synthesis, transport, and metabolism of Secondary metabolites biosynthesis accounted for 3.29%. F1–F2 had the largest DEGs proportion (R-General functional prediction only), accounting for 7.51%, followed by Modification and transport after transcription and protein translation, accounting for 5.63% (Fig. 5).

### KEGG pathways enrichment analysis of DEGs

**Figure 6:**
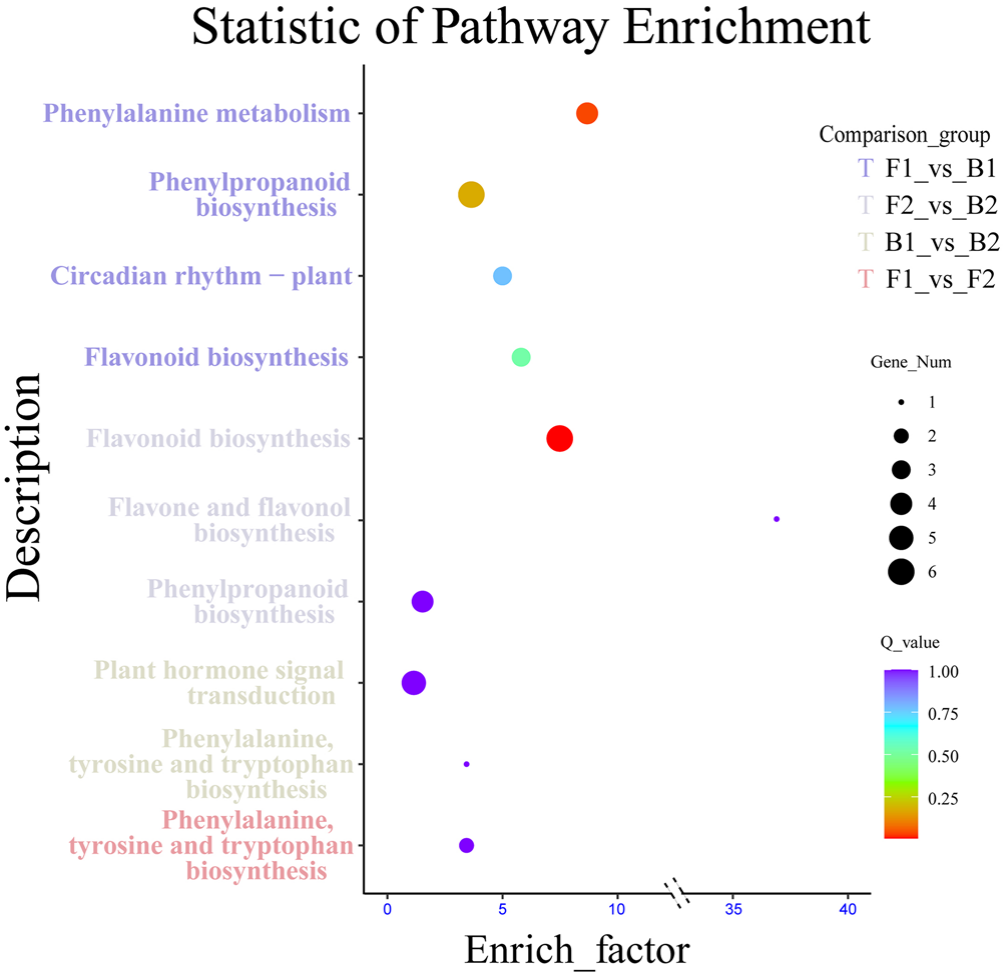
Pathway enrichment analysis of DEGs. The ordinate represents the pathway entry, and the abscissa represents the rich factor. The dots represent the number of significant DEGs, and the significance increases as the dots increase in size. The dots with different colors indicate different Q-values. (The figures were created by R version 4.0.3 based on the KEGG pathway database www.kegg.jp/kegg/kegg1.html).

In order to understand the biological functions of DEGs discussed above, the transcriptome sequencing results were compared with the KEGG public database of tetraploid cultivated peanut and diploid wild peanut. The results showed that 363 DEGs (28.52% of all differential genes) were annotated in the KEGG database. Among them, the metabolic pathways associated with testa color enrichment in F1–B1 included phenylalanine metabolism, phenylpropanol biosynthesis, flavonoid biosynthesis, and plant circadian rhythm. Testa color-related enrichment metabolic pathways in F2–B2 included phenylpropanol biosynthesis, flavonoids and flavonol biosynthesis, and flavonoid biosynthesis. Testa color-related enrichment metabolic pathways in B1–B2 included plant hormone signals transduction and biosynthesis of phenylalanine, tyrosine and tryptophan. Testa color-related enrichment pathways in F1–F2 included phenylalanine, tyrosine, and tryptophan biosynthesis. The diploid and tetraploid reference genomes were compared based on different parts of the same period and different periods of the same part and six metabolic pathways related to anthocyanin biosynthesis were screened out, including phenylpropane acid metabolism, phenylpropanol biosynthesis, flavonoids and flavonol biosynthesis, flavonoid biosynthesis, plant hormone signal transduction, and circadian rhythm in plants. (Fig. 6, Table S6).

### Structural genes related to testa pigment synthesis

**Figure 7:**
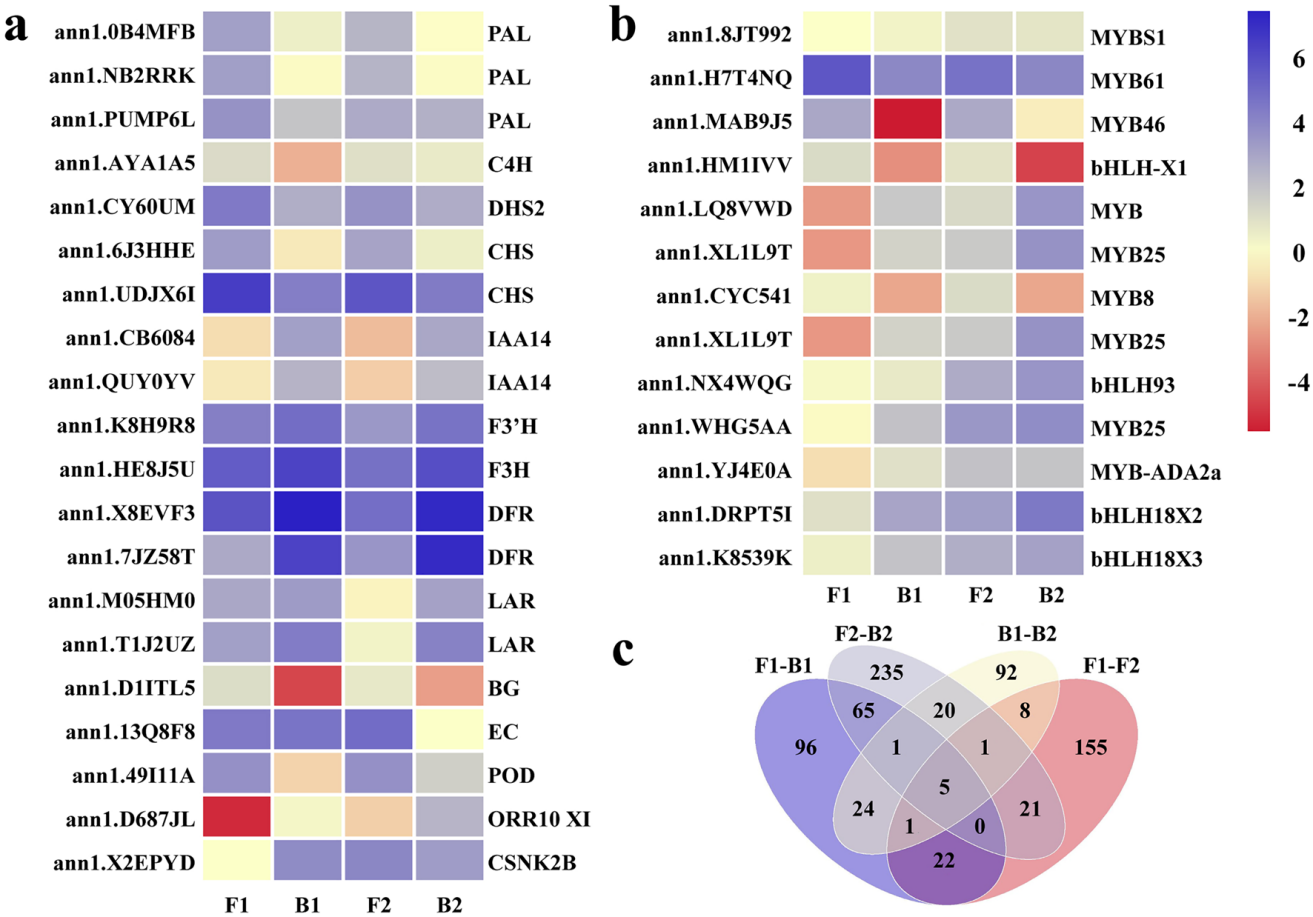
(**a**, **b**) Heat map of the DEGs related to anthocyanin synthesis in the variegated peanut testa among different comparison groups of structural genes and transcription factors. Gene expression was scaled in this analysis using FPKM (Fragments Per Kilobase of exon model per Million mapped fragments) Z-scores based on the mean value of two biological replicates in the heatmap. The key is located on the right-hand side in each case with FPKM values increasing from blue to orange. (**c**) The Venn diagram of the DEGs among different comparison groups.

The Venn diagram analysis showed that 71 DEGs were co-expressed between F1–B1 and F2–B2, 143 DEGs presented specifically in F1–B1, and 277 DEGs were particularly expressed in F2–B2 (Fig. 7c, Table S7). Fifteen DEGs were co-expressed between B1–B2 and F1–F2, 137 DEGs presented specifically in B1–B2, and 198 DEGs were primarily expressed in F1–F2 (Fig. 7c, Table S7). KEGG analysis showed that both F1–B1 and F2–B2 involved differential genes related to peanut testa color and the expression of DEGs was closely related to testa development. Genes involved in the pigment synthesis included 3 *PAL*, 1 *C4H*, 2 *CHS*, 1 *F3H*, 1 *F3'H*, 2 *DFR*, 2 *LAR*, and 2 *IAA* (Table 1). Three *PAL*, 1 *C4H*, 2 *CHS*, and 1 *F3'H* in the non-pigmented areas were down-regulated compared with the pigmented areas. Two *DFR*, 2 *LAR*, and 2 *IAA* in non-pigmented areas were up-regulated compared with the pigmented areas (Fig. 7a), involving the entire anthocyanin metabolism pathway key enzyme gene metabolic pathway.

**Table 1:** Genes related to anthocyanin synthesis in the variegated peanut testa among different comparison groups.

Annotation	Gene ID F1–B1	F1 FPKM	B1 FPKM	FDR	log2FC	Regulated
PAL	arahy.Tifrunner.gnm1.ann1.0B4MFB	9.05	1.45	7.31E − 04	− 2.72	down
PAL	arahy.Tifrunner.gnm1.ann1.NB2RRK	9.63	0.92	8.56E − 11	− 3.47	Down
PAL	arahy.Tifrunner.gnm1.ann1.PUMP6L	12.60	3.96	4.95E − 03	− 1.74	Down
C4H	arahy.Tifrunner.gnm1.ann1.AYA1A5	2.32	0.26	5.48E − 03	− 3.07	Down
DHS2	arahy.Tifrunner.gnm1.ann1.CY60UM	22.02	6.74	3.17E − 03	− 1.77	Down
CHS	arahy.Tifrunner.gnm1.ann1.6J3HHE	10.01	0.68	5.04E − 10	− 3.92	Down
CHS	arahy.Tifrunner.gnm1.ann1.UDJX6I	91.99	20.08	1.65E − 05	− 2.26	Down
IAA14	arahy.Tifrunner.gnm1.ann1.CB6084	0.55	9.01	4.54E − 09	3.96	Up
IAA14	arahy.Tifrunner.gnm1.ann1.QUY0YV	0.68	5.80	1.56E − 04	3.02	up
CSNK2B	arahy.Tifrunner.gnm1.ann1.X2EPYD	0.00	14.94	8.91E − 43	Inf	Up

Annotation	Gene ID F2–B2	F2 FPKM	B2 FPKM	FDR	Log2FC	Regulated
PAL	arahy.Tifrunner.gnm1.ann1.NB2RRK	5.90	0.92	8.56E − 11	− 3.47	Down
F3'H	arahy.Tifrunner.gnm1.ann1.K8H9R8	11.11	26.27	5.59E − 03	1.20	Up
F3H	arahy.Tifrunner.gnm1.ann1.HE8J5U	27.03	62.10	9.35E − 03	1.16	Up
DFR	arahy.Tifrunner.gnm1.ann1.X8EVF3	29.29	152.10	1.81E − 05	2.33	Up
DFR	arahy.Tifrunner.gnm1.ann1.7JZ58T	11.70	144.98	4.15E − 08	3.60	Up
LAR	arahy.Tifrunner.gnm1.ann1.M05HM0	0.81	8.85	8.14E − 13	3.27	Up
LAR	arahy.Tifrunner.gnm1.ann1.T1J2UZ	1.27	18.35	2.87E − 08	3.85	Up
BG	arahy.Tifrunner.gnm1.ann1.D1ITL5	1.70	0.19	9.04E − 04	− 3.16	Down
EC	arahy.Tifrunner.gnm1.ann1.13Q8F8	30.51	0.00	2.99E − 32	#NAME?	Down
POD	arahy.Tifrunner.gnm1.ann1.49I11A	12.86	3.04	2.45E − 07	− 2.12	Down
ORR10 X1	arahy.Tifrunner.gnm1.ann1.D687JL	0.44	5.66	8.33E − 08	3.52	Up
IAA14	arahy.Tifrunner.gnm1.ann1.CB6084	0.55	9.01	4.54E − 09	3.96	Up
IAA14	arahy.Tifrunner.gnm1.ann1.QUY0YV	0.68	5.80	1.56E − 04	3.02	Up

### Analysis of transcription factors differentially expressed in the transcriptome

The transcription factors in the sequencing results were analyzed and it was found that nine differentially expressed *MYBs* were screened from 410 *MYBs* transcription factors, four differentially expressed *bHLHs* were screened from 278 *bHLH* transcription factors, and there were no differentially expressed WD40 family transcription factors (Fig. 7b). All *MYB* regulatory factors were enriched in the two metabolic pathways of splicing and plant circadian transduction. All *bHLH* regulatory factors were also enriched in the two metabolic pathways of plant hormone signal transduction and plant circadian rhythm. However, the differentially expressed *MYB* and *bHLH* transcription factors were not annotated clearly among the metabolic pathways, and the regulatory role of these in the metabolic pathway was unclear (Table S8).

### Joint analysis of transcriptome and metabolome

**Figure 8:**
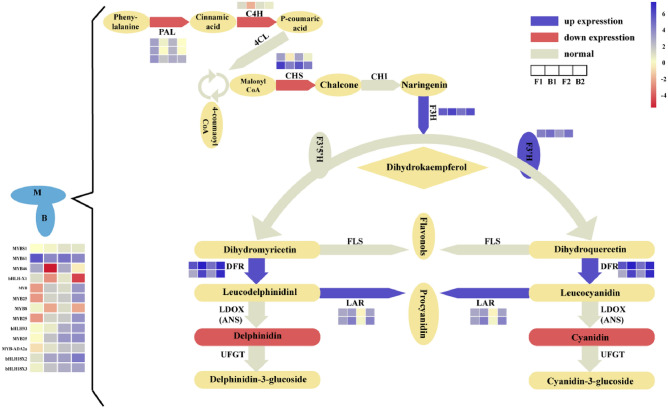
Combined analysis of transcriptome and metabolome of flavonoid biosynthesis. The red graphic represents the down-regulation of gene expression, the blue graphic represents the up-regulation of gene expression, and the gray graphic indicates that there is no significant difference in gene expression.

The combined analysis of metabolome and transcriptome showed that the differential genes in the flavonoid biosynthetic pathway are directly related to the synthesis of delphinidin and cyanidin. The F1–B1 correlation results showed a higher delphinidin and cyanidin content in the pigmented areas compared with the non-pigmented areas. Concomitantly, FPKM (Fragments Per Kilobase of exon model per Million mapped fragments) of 2 *CHSs* and 1 *C4H* increased in the pigmented areas. The F2–B2 correlation results showed that the FPKM of 2 *DFRs*, 1 *F3'H*, 1 *F3H*, and 2 *LARs* in non-pigmented areas increased, and different metabolites resulted in the highest procyanidin content in the non-pigmented areas (Fig. 8).

### qPCR analysis of differential gene expression levels

**Figure 9:**
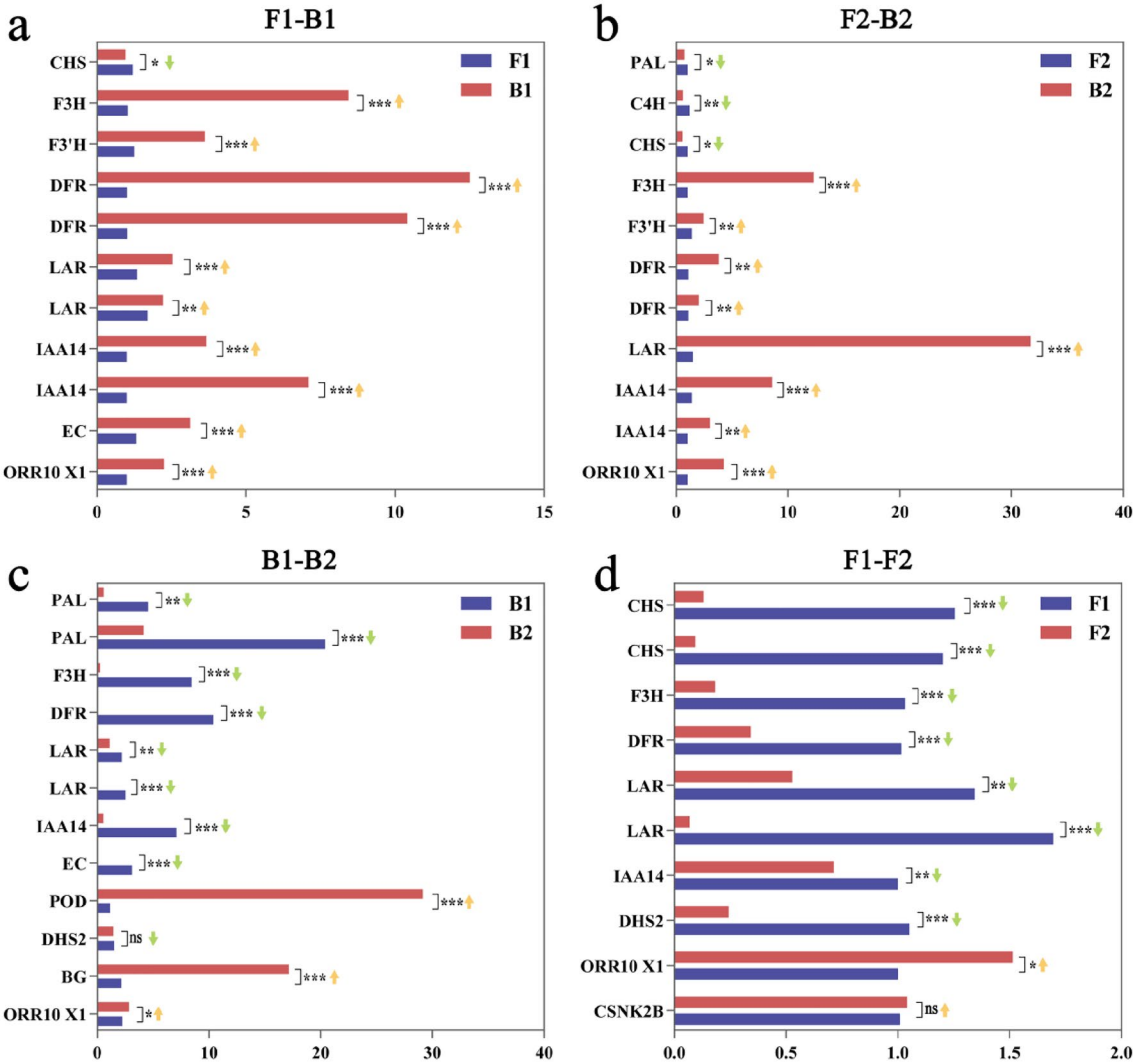
DEGs qRT-PCR verified results. (**a**–**d**) represent F1–B1, F2–B2, B1–B2, and F1–F2, respectively. The x-axis shows the relative gene expression levels analyzed by qRT-PCR. *p < 0.05; **p < 0.01; and ***p < 0.001. ↑ means that the gene is up-regulated in the transcriptome, ↓ the gene is down-regulated in the transcriptome.

Fluorescence quantitative qPCR verification was performed on 20 genes related to anthocyanin metabolism. The results showed that 11 genes were verified in F1–B1 and F2–B2 (Fig. 9a,b), 12 genes were verified in B1–B2 (Fig. 9c), and ten genes were verified in F1–F2 (Fig. 9d), consistent with transcriptome results. The 20 selected differential genes in the four comparison groups showed similar qPCR expression trends to the transcriptome detection results (Fig. 9).

## Discussion

Peanut is one of the five largest oil crops in the world, containing testa that is enriched with biologically active substances. Peanut testae have significant differences in color, with white, red, purple, pink, and variegated colors, of which the variegated testa is unique. Anthocyanin is one of the key substances responsible for color variation in plants^32^. The ingenious sampling is where this research differs from previous studies. In this study, accession VG-01 material with a variegated testa was used, allowing for direct comparisons of mechanisms underlying the color variation within the same genetic background. Because the genetic background of the samples is identical, measurement of the relative flavonoid metabolites can also represent the difference between the pigments in the pigmented and non-pigmented areas. At the same time, the sequencing results were compared and analyzed to both diploid wild and tetraploid cultivated peanuts. Moreover, the molecular regulation mechanism of anthocyanin synthesis has not been previously found in the variegated peanut testa, so this research is of significance to the field of research.

### CHS of the circadian pathway determines the early differentiation of peanut testa color

The synthesis of pigments in the pigmented and non-pigmented areas is comprehensively regulated by multiple structural genes in the variegated peanut testa. The analysis results showed that two key *CHS* genes are the first to regulate pigment synthesis. The up-regulated expression of *CHS* provides a large amount of substrate for the metabolism of anthocyanins downstream of the pigmented areas of variegated testa. Deng et al*.* identified 10 *CHS* genes that were significantly up-regulated, using materials of different colors and different parts on the same wild banana peel^33^. Gao et al*.* adopted transcriptome sequencing to screen *RsCHS- B2* and found that *RsCHS* is up-regulated in red radishes when they formed fleshy roots, which were important genes for red color^34^. Wan et al*.* studied the increase in *CHS* expression during the growth process from DAF20d to DAF60d, which promoted the color accumulation in the peanut testa^22^. The significant difference in our study was that two *CHS* enriched in plant circadian metabolic pathways were identified. We hypothesize that the two *CHS* may be key genes for the color differentiation between the peanut mottling and non-pigmented areas in the early testa development. The F1–B1 correlation results showed that higher delphinidin and cyanidin content in the pigmented areas compared with the non-pigmented areas resulted in the red color of the variegated testa due to the up-regulation of the two *CHS* genes.

### Up-regulated genes in the flavonoid synthesis pathway jointly promote the accumulation of pigments

In the flavonoid synthesis pathway at DAF30, three *PAL*, one *C4H*, and two *CHS* genes are all up-regulated in the pigmented areas, which provides a preliminary product for pigment accumulation in the pigmented areas. Wan et al*.* analyzed the pink peanut testa ZH16 and the results showed that the expression of PAL, 4CL, CHS, CHI, and other enzymes increased with the peanut testa growth^22^. Previous studies reported that *F3H*, *F3'H*, and *F3′5'H* could catalyze the synthesis of three dihydroflavonols (DHK, DHQ, and DHM), where DHK eventually produced brick red geranium pigment, DHQ produced red cyanidin glycosides, and DHM produced blue to purple delphinidin glycosides^35^. In the process of flower color synthesis in dahlia, the expression of *F3H* is significantly down-regulated, changing the color from orange to light red, or even white^36^. Interestingly, we observed up-regulation of *F3H* and *F3'H* in the non-pigmented areas at DAF45. This may be due to the up-regulation of downstream genes in the flavonoid synthesis pathway and that the reverse regulation promotes the increase of *F3H* and *F3′H* expression. Previous studies have shown that silencing the endogenous gene *F3′H* with RNA interference technology could increase the delphinidin content and make the petals of chrysanthemums appear a new type of blue^37^. Therefore, the pigment accumulation pattern of variegated peanut testa may be similar to the studies cited above.

### Procyanidin expressing high content as pre-products accumulated in pigmented areas

*DFR* is the first key downstream gene that affects the anthocyanin synthesis branch in the variegated testa. High expression of *DFR* is advantageous to the accumulation of plant anthocyanins. For example, brick red petunidin 3-O-glucoside can be obtained if *DFR* is introduced into white gerbera^38,39^. Zhang et al*.* studied the up-regulation of *DFR* gene expression in purple leaves in mustard, which contributes to the accumulation of anthocyanins^31^. Different from previous studies, the expression levels of two *DFR* selected by the transcriptome in our study were higher in the non-pigmented areas than in the pigmented areas. The metabolomic analysis determined that the content of Delphinidin and Cyanidin in the pigmented areas was significantly higher than that in non-pigmented areas. There are two possible explanations for the up-regulation of *DFR* in the non-pigmented areas. One is that there is no significant correlation between DFR enzyme activity and testa anthocyanin accumulation during pigment synthesis. Wang et al*.* have reported that DFR enzyme activity in the pericarp was not closely related to anthocyanin synthesis in litchi^40^. Furthermore, there is no significant correlation detected between anthocyanin content and DFR enzyme activity in sweet cherry^41^. The second reason for the significant up-regulation of *DFR* expression in the non-pigmented areas may be that the up-regulated expression of *LAR* causes the leuco anthocyanins to accumulate to procyanidin in the testa. The leuco anthocyanidins are synthesized and accumulated in vacuoles under the catalysis of LAR^42^.

### Hormone inhibition of pigment accumulation in the pigmented areas of variegated peanut testa

Hormones have an antagonistic effect on the synthesis of pigments. The transcription factors bHLH and MYB indirectly regulate the synthesis of anthocyanins by regulating the synthesis of hormones. The results showed that four differentially expressed bHLH and nine MYB regulatory genes were not clearly annotated in the metabolic pathway. It is speculated that bHLH and MYB regulatory genes in the plant circadian rhythm pathway may promote the synthesis of *CHS* to facilitate the accumulation of metabolites in the early stage and make the pigmented areas darker. The synthesis of testa pigment was indirectly regulated by hormones in the plant signal transduction pathway. Wan et al*.* identified 16 DEGs related to plant hormone signal transduction, four of which were down-regulated by the AUX signal and concluded that hormones had an antagonistic effect on pigment synthesis^22^. In this study, we found that the expression levels of 2 *IAA14* hormone-regulated genes in the non-pigmented areas at DAF30 and DAF45 were all up-regulated, which is consistent with previous studies.

### Joint analysis and mutual verification of pigment accumulation

The results of the combined analysis of the transcriptome and metabolome data were corroborated. We found that the metabolic pathway was the most closely integrated pathway of flavonoid biosynthesis, and the nine most closely integrated genes are all key genes in the anthocyanin metabolic pathway. This suggested that the selected 2 *CHS*, 1 *C4H*, 2 *DFR*, 1 *F3'H*, 1 *F3H*, and 2 *LAR* were the key genes for anthocyanin synthesis in peanut. Two different metabolites, Delphinidin and Cyanidin, were enriched in this pathway. Wan et al*.*, in a metabolomic study, showed that flavonoids were redirected in WSC, while multi-omics analyses of WSC mutant seeds and testae demonstrated the influence of WSC on flavonoid biosynthesis in the testa^43^. Kovinich et al*.* found that UGT78K1 and 19 other anthocyanin, (iso)flavonoid, and phenylpropanoid isogenes were differentially expressed, resulting in different testa colors of black (iRT) and brown (irT) soybean (*Glycine max*)^44^, which is consistent with the results of our study. In this study, the transcriptome and metabolome of the pigmented and non-pigmented variegated testa were closely integrated. Following analysis, it was determined that delphinidin and cyanidin were the key pigments that make the pigmented areas color different from that in the non-pigmented areas.

In summary, the identified DEGs in this study were involved in 96 metabolic pathways. Phenylalanine metabolism, phenylpropanol biosynthesis, flavonoids and flavonol biosynthesis, flavonoid biosynthesis, plant hormone signal transduction, and circadian rhythm plants were related pigment synthesis in variegated testa. During the testa development, 14 main structural enzyme genes and 13 regulatory genes affected the pigment synthesis, including phenylalanine ammonia-lyase, cinnamic 4-hydroxylase enzyme, chalcone synthase, dihydroflavonol-3-hydrogenase, dihydroflavonol-3′-hydrogenase, dioxyflavonol-4-reductase, leuco anthocyanins, four bHLHs, and nine *MYB* regulatory genes. Cyanidin and delphinidin were the primary metabolites that caused the color differences between the pigmented and the non-pigmented areas.The results in this study reveal the molecular regulation mechanism of pigment synthesis in peanut testa, which is of great significance in breeding and planting resource protection.

## Materials and methods

### Materials

The variegated testa (accession VG-01) is composed of two parts, the non-pigmented areas (white) and the pigmented areas (red). The seeds were provided by the Peanut Breeding Laboratory of Hebei Agricultural University in P. R. China. VG-01 was flowering and needling on May 6, 2018, and was marked by the hanging thread method at 30, 35, 40, 45, 50, and 55 days after flowering and needling (DAF). 0.6 g variegated testa were used for RNA extraction which was repeated twice.

### Characterization by stereomicroscope and chromatic aberration value

The peanut testa after needling for different days was sliced and the color changes were observed using the stereomicroscope (Olympus SZ61, Japan). The chromatic aberration value measurements result were repeated in triplicate. Different testa parts at different periods were taken, and “L”, “a”, and “b” values were measured using a colorimeter (Konica Minolta Colorimeter CR-10Plus), where “L” represents the lightness value, ”a” represents red and green values, and “b” represents yellow and blue value. F1 and F2 represent the sample names of the pigmented areas at DAF30 and DAF45, respectively. B1 and B2 represent the sample names of the non-pigmented areas at DAF30 and DAF45, respectively.

### Flavonoids extraction from variegated testa

Peanut testa in different pigmented areas at DAF30 and DAF45 were stored at − 80 ℃. Samples were crushed using a mixer mill (MM 400, Retsch) with one zirconia bead (1.5 min, 30 Hz). One-hundred mg of powder was weighed and extracted overnight at 4 ℃ with 1.0 mL of 70% aqueous methanol. Following centrifugation at 10,000*g* for 10 min, the extracts were absorbed (CNWBOND Carbon-GCB SPE Cartridge, 250 mg, 3 mL; ANPEL, Shanghai, China) and filtrated (SCAA-104, 0.22 μm pore size; ANPEL, Shanghai, China) before liquid chromatography-tandem mass spectrometry (LC–MS) analysis.HPLC Conditions: HPLC: column, Waters ACQUITY UPLC HSS T3 C18 (1.8 µm, 2.1 mm*100 mm); solvent system, water (0.04% acetic acid): acetonitrile (0.04% acetic acid); gradient program,100:0 V/V at 0 min, 5:95 V/V at 11.0 min, 5:95 V/V at 12.0 min, 95:5 V/V at 12.1 min, 95:5 V/V at 15.0 min; flow rate, 0.40 mL/min; temperature, 40 ℃; injection volume: 5 μL.

### Species and quantitative analysis of flavonoids in variegated peanut testa

LC–MS/MS was adopted to qualitatively and quantitatively detect the different areas of the variegated testa. The testa was dissolved in dimethyl sulfoxide or methanol and stored at − 20 ℃, which was diluted with 70% methanol to different gradient concentrations before mass spectrometry analysis with ultra-high-performance liquid chromatography and tandem mass spectrometry (MS/MS) (Applied Biosystems 4500 QTRAP^45,46^, were used to qualitatively analyze the primary and secondary spectral data detected by mass spectrometry. Analyst 1.6.3 software was used to analyze the detected data. MultiaQuant software was used to integrate and calibrate the mass spectrometry results to finally obtain the different metabolites.The size of the peak area represented the relative content of metabolites.Two biological replicates and three technical replicates were adopted for each sample.

### Transcriptome sequencing and DEGs analysis

The samples were sequenced and analyzed using the Illumina sequencing platform. The original data sequencing results were compared and analyzed with the diploid wild (Araip. K30076. a1. m1&&Aradu. V14167. a1. M1, Peanutbase) and Tifrunner genomes (http://peanutbase.org/datahypogaea/Tifrunner.gnm1.ann1.CCJH/) and functional annotations were performed. The fragments per kilobase of transcript sequence per million base pairs (FPKM) values in four samples were calculated and DEseq software was used to perform DEGs analysis. Fold Change ≥ 2 and FDR < 0.01 were chosen as DEGs screening criteria. The Benjamini–Hochberg was used to correct the significance *p*-value obtained from the original hypothesis test to reduce the false positive rate, and FDR was used as the key indicator for DEGs screening.

### GO and KEGG analysis of DEGs

GO and KEGG pathway enrichment analysis of the DEGs was based on the standard of p-value < 0.05 and FDR ≤ 0.05. The GO predicted protein information was obtained when the similarity > 30%, E-value < Ie − 5 using the BLAST comparison tool. The metabolic synthesis pathway was annotated by KEGG^47^ (http://www.genome.jp/kegg/pathway.html), which was obtained when compared with the KEGG database under the condition of E-value < le3.

### Joint analysis of transcriptome and metabolome

The differential genes and metabolites screened from the metabolome and transcriptome were jointly analyzed according to the Pearson correlation analysis method. The joint analysis was carried out on the metabolome and transcriptome data based on the standard of the correlation coefficient threshold ≥ 0.8 and correlation P < 0.05.

### Verification by qRT-PCR

The selected 20 DEGs from the pigmented areas were all verified by real-time quantitative PCR (qRT-PCR). Total RNA was extracted by the TRIZOL method, and the corresponding cDNA was synthesized. According to the sequence library required for sequencing, the fluorescent real-time quantitative PCR primers were designed (Table S9) by using Primer Premier5.0 software, in which the peanut actin ACT7 gene was used as the internal reference gene. The ΔΔCt data analysis method was used for gene expression analysis and 2^−ΔΔCt^ was used to calculate the relative gene expression^48^. The expression level in the target gene of the control part (B1, B2 non-pigmented areas) was 1, and two replicates were set in this experiment.

### Ethical statement

All experimental research and field studies on this paper complied with relevant guidelines and legislation of Hebei Agricultural University P. R. China. The experimental methods in this study were performed in accordance with relevant guidelines and legislation of the University Committee.

## Supplementary Information

Supplementary Tables S1.

Supplementary Tables S2.

Supplementary Tables S3.

Supplementary Tables S4.

Supplementary Tables S5.

Supplementary Tables S6.

Supplementary Tables S7.

Supplementary Tables S8.

Supplementary Tables S9.
